# Use of *Opuntia ficus-indica* Fruit Peel as a Novel Source of Mucilage with Coagulant Physicochemical/Molecular Characteristics

**DOI:** 10.3390/polym14183832

**Published:** 2022-09-14

**Authors:** Maria Carolina Otálora, Andrea Wilches-Torres, Carlos Rafael Lara, Gabriel Ricardo Cifuentes, Jovanny A. Gómez Castaño

**Affiliations:** 1 Grupo de Investigación en Ciencias Básicas (NÚCLEO), Facultad de Ciencias e Ingeniería, Universidad de Boyacá, Tunja 150003, Colombia; 2 Grupo Gestión de Recursos Hídricos, Facultad de Ciencias e Ingeniería, Universidad de Boyacá, Tunja 050030, Colombia; 3 Grupo Química-Física Molecular y Modelamiento Computacional (QUIMOL^®^), Escuela de Ciencias Químicas, Universidad Pedagógica y Tecnológica de Colombia, Sede Tunja, Avenida Central del Norte, Tunja 050030, Colombia

**Keywords:** *Opuntia ficus-indica*, mucilage, natural coagulant, wastewater, water treatment

## Abstract

The peels obtained as a byproduct from the processing of fruits (prickly pears) of the Cactaceae family are a rich source of mucilage, a hydrocolloid biopolymer that may have potential application in water/wastewater treatment as a natural coagulant. In this study, the structural (UPLC-QTOF-MS, FTIR, Raman, NMR, XRD, and zeta potential), morphological (SEM), and thermal (DSC/TGA) characterizations of the mucilage extracted from the peels of *Opuntia ficus-indica* (OFI) fruits were carried out. UPLC-QTOF-MS results revealed the presence of a branched polymer with an average molecular weight of 0.44 KDa for this mucilage in aqua media. The NMR spectra of mucilage in DMSO-*d6* indicated that it seemed well-suited as a coagulant with its typical oligosaccharide structure. FTIR studies confirmed the presence of hydroxyl and carboxyl functional groups in the mucilage, indicating its polyelectrolyte nature that could provide coagulating properties through binding and adsorption mechanisms. Likewise, the zeta potential of −23.63 ± 0.55 mV showed an anionic nature of the mucilage. Power XRD technique evidenced the presence of crystalline poly(glycine-β-alanine), glutamic acid, and syn-whewellite. SEM images revealed an irregular and amorphous morphology with cracks, which are suitable characteristics for adsorption mechanisms. The mucilage exhibited two endothermic transitions, with a decomposition temperature in uronic acid of 423.10 °C. These findings revealed that mucilage obtained from OFI fruit peels has molecular and physicochemical characteristics that are suited to its possible application as a natural coagulant in water/wastewater treatments.

## 1. Introduction

Wastewater treatment for domestic and industrial use through chemical coagulation processes that use conventional inorganic/synthetic coagulants, such as aluminum sulfate (Al_2_(SO_4_)_3_) and polyaluminum chloride (PAC), has been in place for many years due to its low cost and widespread availability [1]. However, its use carries some potential risks for the environment, and has been associated with some degenerative diseases in humans [2]. Coagulation processes seek to remove impurities (suspended particles and colloids) in the water by destabilizing them and agglomerating them into larger aggregates by neutralizing the forces that keep them apart. This allows the aggregates, i.e., flocs, to settle down quickly and subsequently be physically separated from the water [3].

Natural polymers derived from agricultural by-products have been proposed as sustainable alternatives for traditional coagulants and coagulation helpers due to their renewal capacity, biodegradability, lower sludge production, non-toxicity, and good profitability (sludge management and coagulant cost) [4]. The polymeric structures of natural coagulants with charged functional groups feature charge neutralization and hydrogen bonding as the two dominant mechanisms for floc formation and impurity removal [4]. In particular, polysaccharide-based coagulants, such as mucilage, have attracted considerable attention for use in water and wastewater treatment because of their significant ability to remove turbidity, dissolved solids, dyes, and chemical oxygen demand (COD).

*Opuntia ficus-indica* (OFI) is a Cactaceae plant that is widely distributed in Central America and most arid areas worldwide [5]. The mucilage extracted from OFI cladodes has been extensively evaluated in coagulation/flocculation processes. Pichler et al. [6] showed that the gelling extract of OFI is a better flocculant than Al_2_(SO_4_)_3_, and that it can be used in water treatment. Likewise, Torres et al. [7] determined that the gelling extract of OFI produces less sludge than FeCl_3_, and that it can be used in the decontamination of municipal wastewater. Bustillos et al. [8] investigated the effect of OFI mucilage treatment on turbidity and COD removal of industrial wastewater. Bouatay and Mhenni [9] demonstrated that OFI mucilage has a better flocculant performance than commercial flocculants, such as EPENWATE EXP31/1 and polyacrylamide A100PWG, in the remediation of textile wastewater obtained from the fabric dyeing and finishing unit DENIM from Tunisia. Mounir et al. [10] determined that mucilage and pectin from OFI can replace inorganic and synthetic flocculants in removing contaminants from synthetic turbid water. DeSouza et al. [11] examined the use of OFI extracts as natural coagulants to remove turbidity and COD from textile effluents compared to FeCl_3_.

Despite its effectiveness, the industrial scaling of mucilage extracted from OFI cladodes as a natural coagulant is hardly feasible as a result of the lack of sustainability of the production process of the entire plant. Instead, the mucilage that can be extracted from OFI fruit results in a natural raw material with greater sustainability and potential application in semi-industrial scale coagulation/flocculation processes. In Latin America, for instance, the consumption and processing of OFI fruit results in the accumulation of large amounts of peels, which represents around 30% of the weight of a whole fruit. This by-product is commonly discarded, thus generating a potential risk for the environment as well as socio-economic problems due to its high disposal costs [12].

OFI fruit peel is a repository of bioactive compounds (polyphenols, betaxanthin, betacyanin, and carotenoids), vitamin C, dietary fiber, fatty acids, and polysaccharides, including mucilage [13,14,15]. The OFI-peel mucilage is an anionic polyelectrolyte heteropolysaccharide, consisting of arabinose (34%), galactose (54%), and xylose (10%) as predominant monosaccharides. This mucilage is among the few emerging polysaccharides that can be used to remove around 50% of the turbidity in water treatment by absorbing several water impurities [15,16,17,18].

As far as we know, there are no reports on the detailed physicochemical/spectroscopical characterization of the mucilage extracted from the peel of the fruit of *Opuntia ficus-indica*. Therefore, in this study, we evaluated the structural (UPLC-QTOF-MS, FTIR, Raman, NMR, XRD, and zeta potential), morphological (SEM), and thermal (DSC/TGA) properties of the mucilage obtained from peels of the fruit of *Opuntia ficus-indica*. Our results reveal molecular/structural insights into the biopolymeric constituents of this type of mucilage that make it suitable for use as a natural coagulant in water treatment.

## 2. Materials and Methods

### 2.1. Chemical and Reagents

Acetonitrile (41.05 g/mol, ≥99.9, CAS No 75-05-8) and formic acid (46.03 g/mol, >98%, CAS No 64-18-6) solvents (HPLC grade), as well as ethanol (46.07 g/mol, analytical grade, 97%, CAS No 64-17-5), were purchased from Merck (Darmstadt, Germany). DMSO-d6 (84.17 g/mol, 99.9 atom% D, CAS No 2206-27-1) was purchased from Cambridge Isotope Laboratories, Inc. (Tewksbury, MA, USA).

### 2.2. Plant Material

Freshly cut peels of *Opuntia ficus-indica* fruits were collected from local food restaurants, washed with distilled water at room temperature, and cut into small pieces. OFI fruit peels presented a pH of 5.44 ± 0.02 and soluble solids content of 9.7 ± 0.02 ° Brix, which were determined using a digital pH meter (ORION™ Versa Star™, Thermo Scientific Inc., Waltham, MA, USA) and a digital handheld refractometer (Boeco model 32395, Hamburg, Germany), respectively.

### 2.3. Mucilage Extraction

**Figure 1:**
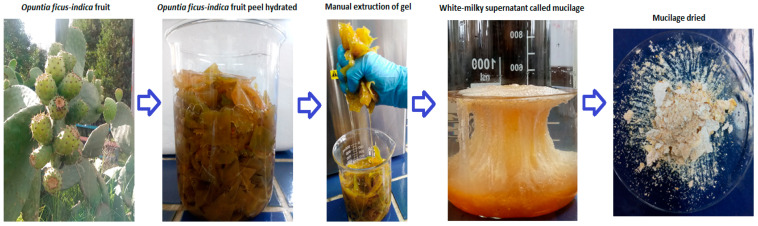
Selected photographs of the process of extracting mucilage from the peels of the fruit of *Opuntia ficus-indica*.

The small pieces of the OFI fruit peels were placed into 100-milliliter beakers to which distilled water was added at room temperature in a 1:2 *w*/*v* ratio (peel:water) and left for 12 h. This process seeks to improve the dissolution rate of the polymeric compounds by enhancing their interactions with the dispersed particles during coagulation/flocculation processes [19]. The hydrated peels were manually squeezed to extract their gel. Then, 95% ethanol at 18 °C was added to the extracted gel in a ratio of 3:1 (ethanol:gel), and the mixture was allowed to stand for 15 min without stirring until the formation of a milky-white supernatant corresponding to the OFI fruit peel mucilage. This treatment removes the oils from the mucilage, which contribute to organic loads [20]. The mucilage was collected and then dried in an oven at 50 °C for 3 h. The dry material was macerated manually in a porcelain mortar and subsequently sieved through a 60-mesh until a fine powder was obtained (standard granulometry ≤ 250 μm). The powdered mucilage was placed in high-density polyethylene bags and stored in a desiccator, at room temperature with a relative humidity of 30%, until characterization. Figure 1 shows photographs of the main stages involved in the process of extracting mucilage from the peels of *Opuntia ficus-indica* fruits.

### 2.4. Mucilage Physicochemical/Molecular Characterization

#### 2.4.1. UPLC-QTOF-MS

The molecular weight average values (Mn, Mw, Mz, and *M_z_*_+1_) and the polydispersity index (Mw/Mn) of the main biopolymeric component of the powdered mucilage were determined using an Acquity H Class plus Ultra High-Performance Liquid Chromatography (UPLC) equipment coupled a with Xevo-G2-XS Quadrupole Time of Flight (QTOF) detector (Waters Corporation, Milford, MA, USA). These UPLC-QTOF analyses were performed in positive electrospray ionization (ESI) mode. A sample of powdered mucilage was diluted in deionized water to a concentration of 1 mg/mL and stirred for 6 h at room temperature. The solution was then centrifuged at 5000 rpm for 15 min and the supernatant was filtered through a 0.45-micron Millipore filter. Aliquots (5 μL) were separated through an Acquity UPLC BEH C18 analytical column (2.1 mm × 100 mm, 1.7 µm particle size). The eluent system was composed of type I water and 0.1% formic acid (solvent A) and acetonitrile and 0.1% acid formic (solvent B) at a flow rate of 0.4 mL/min. The gradient elution program was set as follows: 0–5 min (95% A), 6–18 min (50% A), and 19–20 min (95% A). The following parameters were maintained: source temperature 120 °C, desolvation temperature 350 °C, desolvation gas flow rate at 800 L/h, and cone gas flow rate of 100 L/h. The cone and capillary voltages were set at 20 V and 2.5 kV, respectively.

The number average molecular weight (Mn), which is the statistical average molecular weight of all the polymer chains in the sample, was calculated using Equation (1):

(1) $$Mn=\frac{\sum N_{i}M_{i}}{\sum N_{i}},$$

where $M_{i}$ is the molecular weight of a chain and $N_{i}$ is the number of chains of that molecular weight.

The weight average molecular weight (Mw) and higher average molecular weights (Mz and *M_z_*_+1_) were calculated according to Equation (2):

(2) $$M=\frac{\sum N_{i}M_{i}^{n+1}}{\sum N_{i}M_{i}^{n}},$$

where n=1 gives M=Mw, n=2 gives M=Mz, and n=3 gives M= *M_z_*_+1_.

The polydispersity index (*I*) is used as a measure of the broadness of a molecular weight distribution of a polymer, and is defined by Equation (3):

(3) $$I=\frac{Mw}{Mn},$$

#### 2.4.2. Fourier-Transform Infrared (FTIR) Spectroscopy

The infrared spectra of powdered mucilage were recorded on a Shimadzu Prestige 21 spectrophotometer (Duisburg, Germany) equipped with a Michelson-type interferometer, a KBr/Ge beam-splitter, a ceramic lamp, and a DLATGS detector. The FTIR spectra were measured in the range of 4500–4520 cm^−1^ with a resolution of 3.0 cm^−1^ and 30 cumulative scans, using the attenuated total reflectance/reflection (ATR) technique.

#### 2.4.3. Raman Spectroscopy

The chemical composition of powdered mucilage was analyzed using a Raman spectrophotometer (DXR™ Smart Raman, Thermo Scientific, Waltham, MA, USA) equipped with a 785 nm excitation diode laser. Spectra were measured with an average scan time of 1.0 s using a laser power of 20.0 mW. A total of 20 scans per spectra were performed to improve the signal-to-noise ratio.

#### 2.4.4. Nuclear Magnetic Resonance (NMR)

The powdered mucilage molecular structure was analyzed by one-dimensional (^1^H, ^13^C, and DEPT 135) and bi-dimensional (COSY, HSQC and HMBC) spectroscopy, using a Bruker Avance DPX 250 MHz spectrometer operating at 9.4 Tesla. Approximately 32 mg of sample was solubilized in 0.6 mL DMSO-d6. All analyses were performed at 298 °C, and frequencies of 400.16 MHz and 100.63 MHz were used for ^1^H and ^13^C nuclei, respectively.

#### 2.4.5. X-ray Diffraction (XRD) Analysis

X-ray diffraction analysis of powdered mucilage was performed on a Bruker D8 Advance DaVinci Geometry X-ray diffractometer (Bruker-AXS, Bremen, Germany) with a Lineal LynxEye detector using Cu-Kα radiation, produced at 40 kV and 40 mA. Data were recorded from the range of 4° to 70° (step size 0.02° and 0.6 s counting time for each step).

#### 2.4.6. Zeta Potential

The zeta potential of the mucilage sample was measured using a NanoPlus Particle Size Zeta Potential Analyzer, NanoPlusTM 3” (Norcross, GA, USA) at 25 and pH 5.68. The powdered mucilage (608 mg) was dispersed in 100 mL of distilled water by using a magnetic stirrer (C-MAG HS 7 S000, IKA, Staufen im Breisgau, Germany) at 8000 rpm for 6 h at 18 °C and pH = 7.

#### 2.4.7. Scanning Electron Microscopy (SEM)

The microscopic morphology of powdered mucilage was evaluated via scanning electron microscopy (SEM) using EVO MA 10-Carl Zeiss equipment (Oberkochen, Germany) operating at 20 kV. All samples were coated by gold–palladium sputtering before their examination.

#### 2.4.8. Thermal Analysis

The thermogravimetric analysis (TGA)/differential scanning calorimetry (DSC) of powdered mucilage was performed on a TA Instrument (SDT Q600 V20.9 Build 20, New Castle, DE, USA). Argon was used as a purge gas (100 mL/min). The dried samples of powdered mucilage were placed in aluminum pans and heated from 20 to 600 °C at a heating rate of 10 °C/min.

### 2.5. Statistical Analysis

Except where stated, all experiments were carried out in triplicate ± standard deviation. Data were analyzed using analysis of variance (ANOVA), and means were compared using Fisher’s least significant differences test (*p* < 0.05).

## 3. Results and Discussion

### 3.1. Mucilage in Solution

#### 3.1.1. Polymer Molecular Weight Distribution in Water

**Figure 2:**
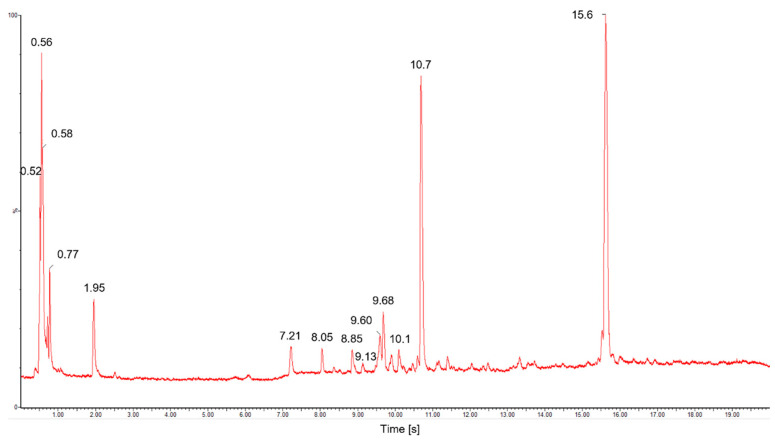
Peak chromatogram for mucilage extracted from *Opuntia-ficus* fruit peels obtained by UPLC-QTOF-MS.

The ultra-high performance liquid chromatography for an aqueous sample of mucilage extracted from OFI fruit peel is shown in Figure 2. This chromatographic separation allowed for the detection of around fifteen water-soluble metabolites within a range of 20 s retention time (r.t), whose molecular ions [M + H]^+^ and relative abundances (in%) are listed in Table S1 in Supplementary Materials.

**Figure 3:**
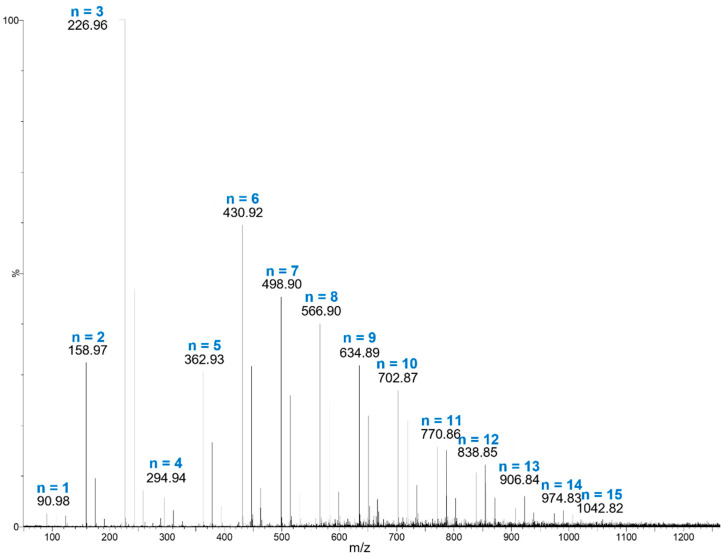
Mass spectrum of the main polysaccharide component of mucilage extracted from OFI fruit peels obtained by UPLC-QTOF-MS.

As shown in Figure 2, three main metabolites contribute around 70% to the total composition of the OFI-fruit-peel mucilage aqueous sample. They were detected at retention times of 0.56, 10.7, and 15.6 min, and with contributions of 14.1, 21.3, and 33.5%, respectively. The rest of the metabolites presented percentage contributions ≤ 5% each (Table S1), and were left unassigned. The main peak at r.t of 15.6 min showed an m/z ratio of 290.28, and was assigned to catechin (C_15_H_14_O_6_), which is a flavonoid metabolite that provides antioxidant activity [21]. The second most abundant component (r.t = 21.3 min) was matched to a mass of C_33_H_36_N_2_O_15_ (*m*/*z* = 700.6), and was tentatively assigned as an unknown betacyanin derivative, which is a class of betalain pigments that are commonly present in cactus fruit peels [13]. The third most abundant component (r.t = 0.56 min) showed a mass spectrum with a Gaussian distribution and a recurring mass loss of 67.99 *m*/*z* (Figure 3), which are typical characteristics of a polymer (polysaccharide).

The mass data extracted from Figure 3 provide the average molecular weight values (*Mn*, *Mw*, *Mz* and *M_z_*_+1_) and polydispersity index (*Mw/Mn*) of the main polysaccharide component of the OFI fruit peel mucilage, which are presented in Table 1. In total, fifteen polymeric chain molecular ions [M + H]^+^, from *N* = 1 to 15, make up this branched biopolymeric sugar system, from which a statistical average molecular weight of 438.80 Da was calculated. This relatively low average molecular weight for a polymer contributes to a better water solubility that facilitates the use of this mucilage as a potential coagulant; this is in contrast to other related biopolymeric compounds, such as chitosan, which have much higher molecular weights (50–2000 kDa) [15,22]. The largest weighted contribution to the average molecular weight in the mucilage polymer from OFI fruit peels comes from chains of length *N* = 3, indicating that this fragment is the most abundant branching/termination in this biopolymer. *N =* 1 gives an *m*/*z* ratio of 91 Da [M + H]^+^ (Figure 3), which led to the identification of the monosaccharide component as C_3_H_6_O_3_, corresponding to a triose (glyceraldehyde). Meanwhile, the loss of a 68-Dalton recurring mass between polymer chains is consistent with carbon suboxide (C_3_O_2_).

A polydispersity index (*I*) of 1.22 was calculated for this biopolymer (Table 1), indicating a relatively low molecular weight distribution width among its chain lengths. The value of this parameter usually ranges between 1.16 and 2.86 for natural polymers [15,22]. The closer the polydispersity index is to unity, the more homogeneous the molecular weight distribution is; e.g., a monodisperse polymer where all chain lengths are equal (such as a protein) has *Mw*/*Mn* = 1.

#### 3.1.2. NMR Spectra of Mucilage in DMSO-d6

^1^D-NMR spectra (^1^H, ^13^C and DEPT/^13^C) and 2D-NMR spectra (COSY, HMBC and HSQC) of a sample of OFI fruit peel mucilage dissolved in DMSO-*d6* are shown in Figures S1–S3 and S4–S6, respectively, in Supplementary Materials.

^1^H-NMR spectrum shows resonances (in ppm) at 1.06 (t, *J* = 7.0 Hz, 3 H), 1.24 (s, 1 H), 2.89 (t, *J* = 8.30 Hz, 1 H), 3.07 (ddt, *J* = 21.6, 17.7, 7.9 Hz, 4 H), 3.25 (s, 1 H), 3.54 (m, 9 H), 3.80 (m, 1 H), 4.27 (dd, *J* = 8.0, 3.3 Hz, 1 H), 4.38 (s, 3 H), 4.49 (s, 3 H), 4.70 (s, 2 H), 4.85 (s, 3 H), 4.91 (s, 1 H), 5.11 (s, 1 H), 6.21 (s, 1 H), 6.58 (t, *J* = 7.8 Hz, 1 H), and 6.93 (m, 1 H). ^1^H-^1^H COSY NMR spectrum: (6.58–6.93), (6.58–4.27), (6.21–4.91), (4.91–3.07), (4.27–2.89), (4.70–3.25), (3.54–1.06).

The ^13^C NMR spectrum shows a distinctive profile for a carbohydrate, with resonances for CH_3_ (δ~20 ppm), CH_2_ (60 ≤ δ ≤ 70 ppm), CH (70 ≤ δ ≤ 90 ppm), C (90 ≤ δ ≤ 110 ppm), and C=C (δ ≤ 125 ppm): 19.01 (CH_3_,), 56.60 (CH_2_,), 61.68 (CH_2_,), 63.40 (CH_2_,), 70.76 (CH), 71.05 (CH), 72.42 (CH), 72.83 (CH), 73.54 (CH), 75.29 (CH), 75.79 (CH), 76.16 (CH), 77.21 (CH), 82.35 (CH), 92.69 (anomeric, CH), 97.36 (anomeric, CH), 98.51 (CH), 102.45 (CH), and 131,70 (C). HSQC 2D-NMR spectrum: (6.93–131.70), (4.91–92.69), (4.27–97.36), (3.80–75.79), (3.54–72.42), (3.07–70.76), (3.07–72.83), (3.07–77.21), (2.89–75.29), and (1.06–19.1). HMBC 2D-NMR spectrum: (1.06–56.6).

### 3.2. Powdered Mucilage

#### 3.2.1. Molecular Vibrations

**Figure 4:**
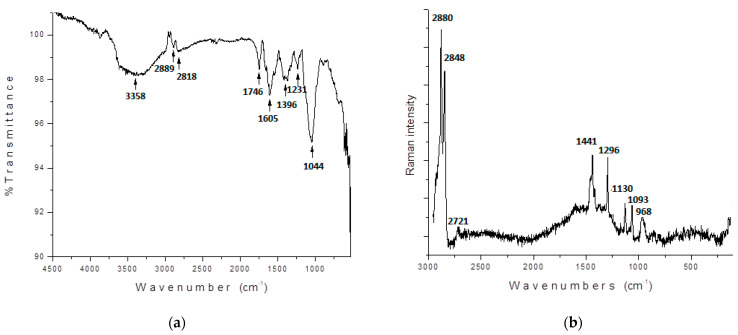
FTIR (**a**) and Raman (**b**) spectra of powdered mucilage extracted from OFI fruit peels.

The experimental FTIR and Raman spectra of the powdered mucilage extracted from OFI fruit peels are shown in Figure 4. The FTIR spectrum of the powdered mucilage (Figure 4a) showed a broad band centered at 3358 cm^−1^ that was attributed to the stretching of the hydroxyl groups (O - H) that adhered both to the polysaccharide structure and to the carboxylic terminals of galacturonic acid. The broad profile of this absorption reflects an extended vibrational distribution caused by many hydrogen-bond (HB) type interactions. The absorptions observed at around 2889 and 2818 cm^−1^ were assigned to the stretching asymmetric and symmetric C-H vibrations, respectively. The absorptions observed in 1746 and 1605 cm^−1^ were assigned to the stretching vibration of carbonyl (C=O) and carboxylic (-COOH) functional groups, respectively. The band observed at 1396 cm^−1^ indicates the presence of C-O in the carboxylic groups which are mainly found in galacturonic acid. The signals at 1231 and 1044 cm^−1^ are characteristic of polysaccharides indicating the presence of different functional groups such as C-O-H and C-O-C bending, respectively [23,24,25].

The FTIR spectrum shows the presence of organic functional groups that give rise to coagulating activity of mucilage. As reported by other studies [24], the presence of the hydroxyl and carboxyl functional groups (that reflects the polyelectrolyte nature of mucilage) in the galacturonic acid structure are responsible for the coagulation process, providing a bridge for the particles to adsorb upon. 

The Raman spectrum of the powdered mucilage (Figure 4b) showed a region between 2880 and 2848 cm^−1^ that was attributed to stretching asymmetric and symmetric C-H vibrations, which correlate with pentose rings in the polysaccharide structure. The signal observed around 1441 cm^−1^ was assigned to the symmetric deformations of CH_3_ in the acetyl groups. The signal at 1090 cm^−1^ indicates the O–H vibrational elongation. The absorption observed at 968 cm^−1^ suggests the presence of β-glycosidic linkage [27,28].

#### 3.2.2. X-ray Powder Diffraction (XRD)

**Figure 5:**
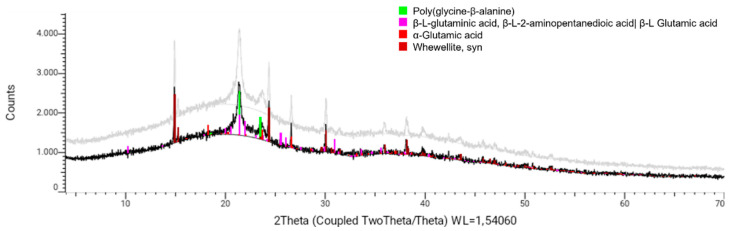
X-ray powder diffraction pattern (black line) from a solid sample of OFI fruit peel mucilage. The gray line is an overlay of the same diffraction pattern for better viewing.

Measurement of the X-ray diffraction pattern of a dry solid sample of mucilage extracted from OFI fruit peels allowed for the identification of four crystalline phases. As seen in Figure 5, these crystal phases coincided with the diffraction patterns recorded for the poly-glycine-β-alanine protein (C_0_._84_H_1_._26_N_0_._42_O_0_._42_(C_3_H_5_NO)_0_._58_), the amino acids β-L-glutaminic acid (C_5_H_9_NO_4_) and α-glutamic acid (C_5_H_9_NO_4_), and the hydrated calcium oxalate (whewellite, CaC_2_O_4_.H_2_O) in its syn form. Other reflections were observed in the X-ray diffraction profile that could not be assigned to known crystalline phases.

#### 3.2.3. Morphological and Thermal Characterizations

**Figure 6:**
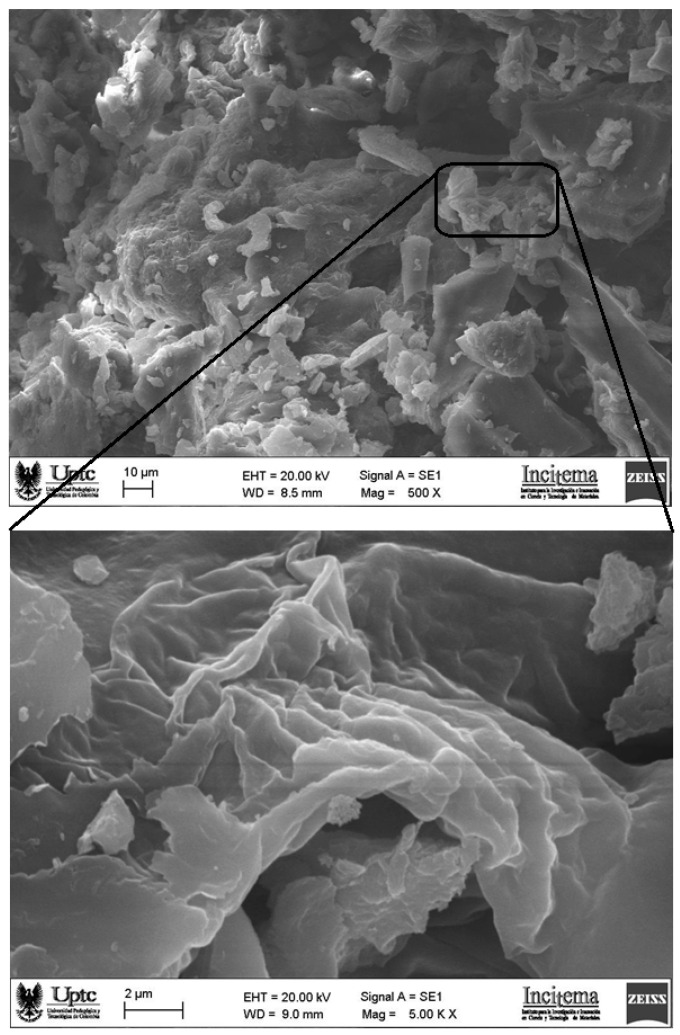
SEM micrograph images of the surface at 500× (**top**) and 5000× (**bottom**) of powdered mucilage extracted from *Opuntia ficus-indica* fruit peel.

**Figure 7:**
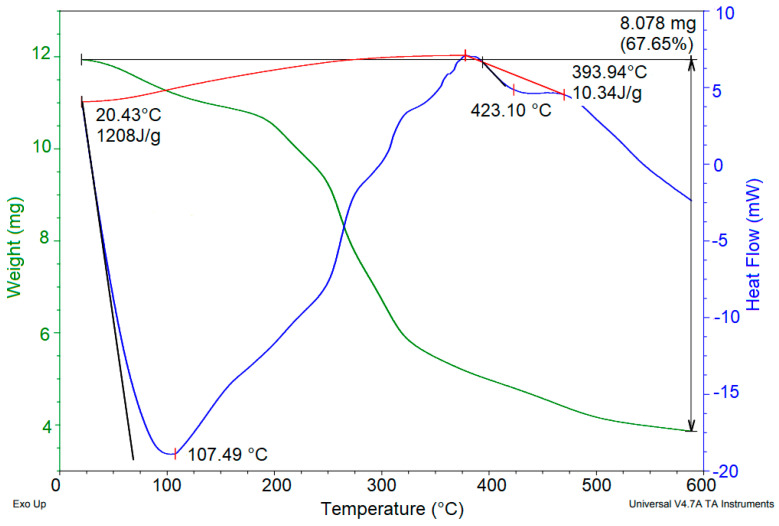
TGA/DSC thermograms of powdered mucilage from *Opuntia ficus-indica* fruit peel.

SEM micrographs and thermal behavior (DSC/TGA) of powdered mucilage extracted from OFI fruit peels are presented in Figure 6 and Figure 7, respectively.

The surface structure of the mucilage, captured at 500× magnification (Figure 6 top), shows a rough, cracked, and porous texture, along with the presence of cavities (adsorption sites) of irregular shape and size. This morphology fits well with adsorption chemistry, that is, with molecular systems that facilitate the adsorption of particles/molecules/ions of different sizes (e.g., wastewater contaminants) [29,30]. Similar morphologies were observed for flaxseed mucilage that was used as a green coagulant in the removal of surfactants from industrial effluents [31], and for the *Opuntia ficus-indica* cladode mucilage that was used as a biocoagulant for pretreating waters affected using bituminous and processes [23]. The images of the mucilage that were taken with a magnification of 5000X (Figure 6 bottom) revealed small particles that possibly corresponded to protein aggregates adhered to the carbohydrate blocks of the sample, which agrees with the results obtained using XRD (Section 3.2.2). The morphology of the mucilage obtained from the shells of the *Opuntia ficus-indica* fruit presents a suitable microstructure for an adsorption mechanism in the coagulation process [32,33].

The thermogram (Figure 7) of the OFI fruit peel mucilage revealed two main endothermic events. The first occurred between 25 and 250 °C (peak 107.49 °C), with a related mass loss of 12.31%. This event was attributed to the loss of adsorbed and structural water within the polysaccharide, followed by gelatinization. The second event occurred between 250 and 475 °C (peak 423.10 °C), with a mass loss of 67.65%. This event was attributed to the degradation of the polysaccharide structure and the subsequent decomposition/volatilization of the material. Uronic acids also decompose at 400–500 °C [34]. Similar thermal behavior was previously reported for *Opuntia dillenii haw* fruit peel mucilage [29]. According to the above, the galacturonic acid content of the mucilage, due to the decomposition temperature range, contributes to the coagulation process through the formation of hydrogen bonds for the adsorption of colloids [23,35].

#### 3.2.4. Zeta Potential

The zeta potential for the mucilage showed a negative charge (−23.63 ± 0.55 mV), whereby the sample is classified as an anionic polyelectrolyte biopolymer. Bouauinea et al. [36] reported a similar zeta potential value (−23 mV) for mucilage extracted from *Opuntia ficus-indica* cactus pads. The anionic nature of the mucilage (-CO and -COOH) suggests that the coagulation behavior of the polysaccharide occurs through an adsorption bridge mechanism, resulting from the dipolar interaction of the mucilage with the divalent cations present in *Opuntia spp*. [37]. In other words, the negative charge of the mucilage is due to the ionization of its functional groups [19].

## 4. Conclusions

The extraction, purification and drying of the mucilage of fruit (prickly pears) peels of *Opuntia ficus indica* were carried out. The physicochemical/spectroscopic characterization of this mucilage was carried out using UPLC-QTOF-MS, FTIR, Raman, NMR, XRD, zeta-potential, scanning electron microscopy (SEM), and DSC/TGA techniques. The mucilage powder presented a yellow coloration, which according to the results of UPLC-QTOF-MS, may be associated with the adhesion of flavonoids and pigments (betalains) to the structure of the main polysaccharide compound. The powder FTIR and Raman spectra, as well as the NMR spectra in DMSO-*d6* of the mucilage fit classical profiles of a polysaccharide; however, the amount dissolved in water related to this polysaccharide could be affected by the sample preparation process employed for the UPLC-QTOF-MS studies. It is very likely that in water, the main polysaccharide component of this mucilage presents opening of the pentose ring cycles, which leads to a structural rearrangement that is reflected in the loss of repeated suboxide units during ESI ionization MS characterization. This biopolymer presented a significantly lower average molecular weight (0.44 Da) than the polysaccharides obtained in other mucilages. According to X-ray diffraction studies, the dried mucilage sample shows some crystalline components identified as poly-glycine-β-alanine protein, β-L-glutaminic and α-glutamic amino acids, and hydrated calcium oxalate (whewellite, CaC_2_O_4_.H_2_O). These crystalline components were associated with small aggregates, segregated from the polymeric matrix, as could be observed through SEM. The SEM micrographs additionally revealed a mucilage morphology dominated by the presence of cracks, pores, and roughness of variable sizes. Likewise, the Z potential of the solid-mucilage sample reflects an ionic condition for this mucilage. In summary, the physicochemical/molecular properties extracted from this study reflect outstanding structural and chemical conditions of mucilage extracted from the discarded peels of *Opuntia ficus indica* fruits, which provide added value to these agricultural bioproducts.

## Figures and Tables

**Table 1:** Molecular weight average values (in Da) and polydispersity index (*I*) of the main biopolymeric component of powdered mucilage extracted from peels of OFI fruits.

*N_i_* Max	*Mn*	*Mw*	*Mz*	*M_z_*_+1_	*I*
15	438.80	536.80	613.95	671.78	1.22

## Data Availability

The data presented in this study are available on request from the corresponding author.

## Supplementary Materials

The following supporting information can be downloaded at: https://www.mdpi.com/article/10.3390/polym14183832/s1, Figure S1: ^1^H NMR spectrum (in DMSO-*d6*) of a mucilage sample extracted from OFI fruit peels; Figure S2: ^13^C NMR spectrum (in DMSO-*d6*) of a mucilage sample extracted from OFI fruit peels; Figure S3: DEPT/^13^C NMR spectrum (in DMSO-*d6*) of a mucilage sample extracted from OFI fruit peels; Figure S4: ^1^H-^1^H COSY NMR spectrum (in DMSO-*d6*) of a mucilage sample extracted from OFI fruit peels; Figure S5: HSQC 2D-NMR spectrum (in DMSO-*d6*) of a mucilage sample extracted from OFI fruit peels; Figure S6: HMBC 2D-NMR spectrum (in DMSO-*d6*) of a mucilage sample extracted from OFI fruit peels; Table S1: [M + H]^+^ ions masses of the main components of mucilage extracted from OFI fruit peels detected by UPLC-QTOF-MS.
