# Supplementary material for: Use of Opuntia ficus-indica Fruit Peel as a Novel Source of Mucilage with Coagulant Physicochemical/Molecular Characteristics
Source: Polymers (Basel). 2022 Sep 14;14(18):3832. doi: 10.3390/polym14183832 (PMC9504202; doi:10.3390/polym14183832)
Supplement: Supplementary file 1 [file polymers-14-03832-s001.zip › polymers-1917295-supplementary.pdf]

polymers

MDPI

Supplementary Materials

Use of *Opuntia ficus-indica* fruit peel as a novel source of mucilage with coagulant physicochemical/molecular

Maria Carolina Otálora [1,*], Andrea Wilches-Torres [1], Carlos Rafael Lara [2], Gabriel Ricardo Cifuentes [2] and Jovanny A. Gómez Castaño [3,*]

[1] Grupo de Investigación en Ciencias Básicas (NÚCLEO), Facultad de Ciencias e Ingeniería, Universidad de Boyacá, Tunja 050030, Colombia
[2] Grupo Gestión de Recursos Hídrico, Facultad de Ciencias e Ingeniería, Universidad de Boyacá, Tunja 050030, Colombia
[3] Grupo Química-Física Molecular y Modelamiento Computacional (QUIMOL®), Escuela de Ciencias Quí-micas, Universidad Pedagógica y Tecnológica de Colombia, Sede Tunja, Avenida Central del Norte, Tunja 050030, Colombia
* Correspondence: marotalora@uniboyaca.co (M.C.O); jovanny.gomez@uptc.edu.co (J.A.G.C)

Citation: Otálora, M.C.; Wilches-Torres, A.; Lara, C.R.; Cifuentes, G.R.; .; Gómez Castaño, J.A. Use of *Opuntia ficus-indica* Fruit Peel as a Novel Source of Mucilage with Coagulant Physicochemical/ Molecular Characteristics. *Polymers* **2022**, *14*, 3832. https://doi.org/10.3390/polym14183832

Academic Editor: Irshad Kammakakam and Mostafa Khodakarami

Received: 30 August 2022
Accepted: 11 September 2022
Published: 14 September 2022

Publisher's Note: MDPI stays neutral with regard to jurisdictional claims in published maps and institutional affiliations.

Copyright: © 2022 by the authors. Submitted for possible open access publication under the terms and conditions of the Creative Commons Attribution (CC BY) license (https://creativecommons.org/licenses/by/4.0/).

Table S1. $[M+H]^+$ ions masses of the main components of mucilage extracted from OFI fruit peels detected by UPLC-QTOF-MS.

R.T (min)	Relative %	$[M+H]^+$	Molecular formula	Identification	Class
0.52	5.2	167.0178	$C_8H_7O_4$	Hydroxybenzoic acid	Polyphenol
0.56	14.1	*	-	Biopolymer	Polysacharide
0.58	3.9	383.1219	-	-	-
1.95	4.3	279.0549	$C_{16}H_{23}O_4$	-	-
7.21	2.4	263.0602	-	-	-
8.05	1.5	371.2734	-	-	-
8.85	1.7	372.2556	-	-	-
9.13	0.6	457.3461	-	-	-
9.60	3.0	757.2263	-	-	-
9.68	3.4	453.3515	-	-	-
9.80	1.3	215.1806	-	-	-
10.1	1.6	598.4278	-	-	-
10.7	21.3	701.5020	$C_{33}H_{36}N_2O_{15}$	Betacyanin derivative	Betalain
15.6	33.5	290.2764	$C_{15}H_{14}O_6$	Catechin	Flavonoid

* See Figure 3 and Table 1 in the main document for mass information.

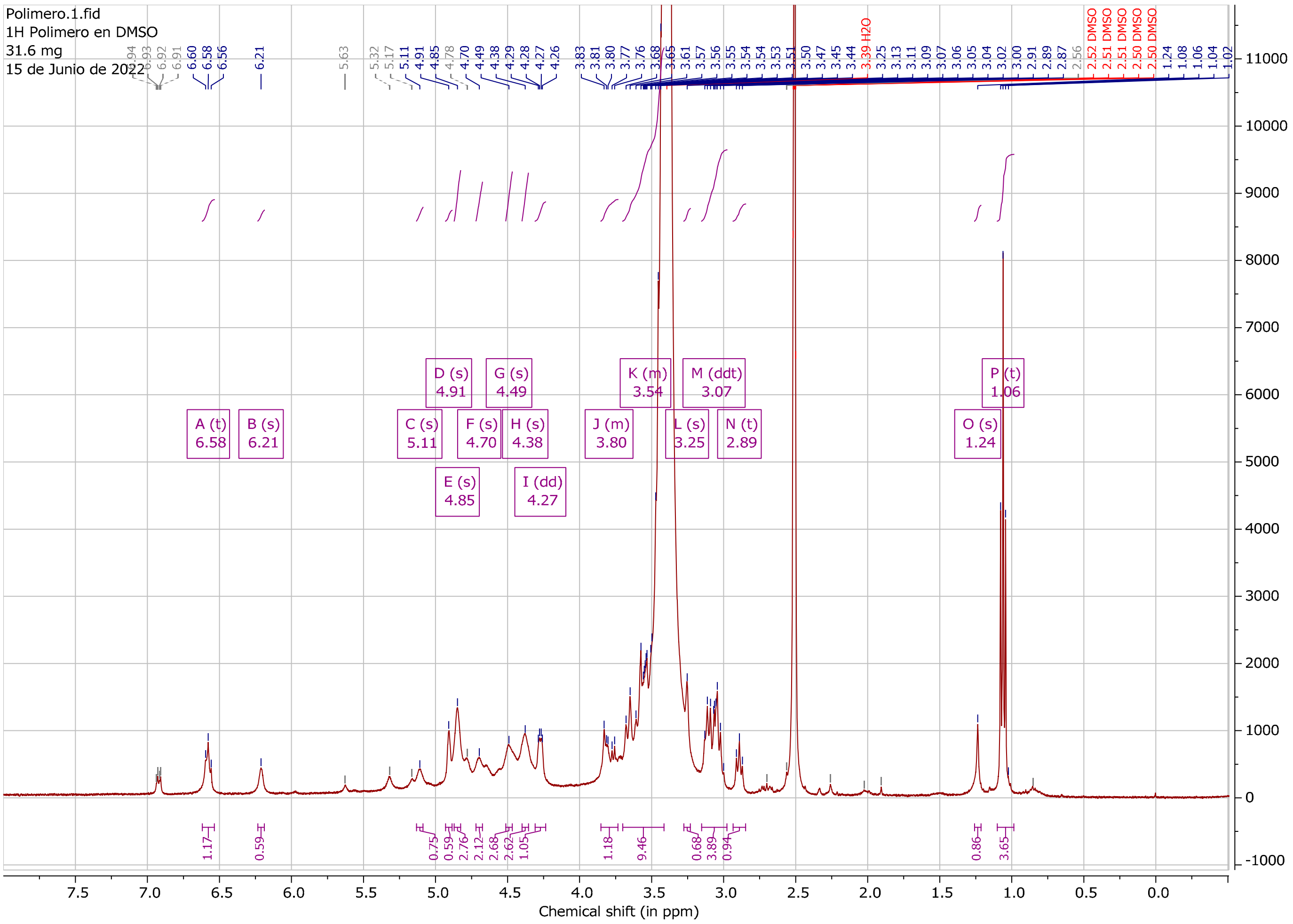

Figure S1. ^{1}H NMR spectrum (in DMSO-*d6*) of a mucilage sample extracted from OFI fruit peels.

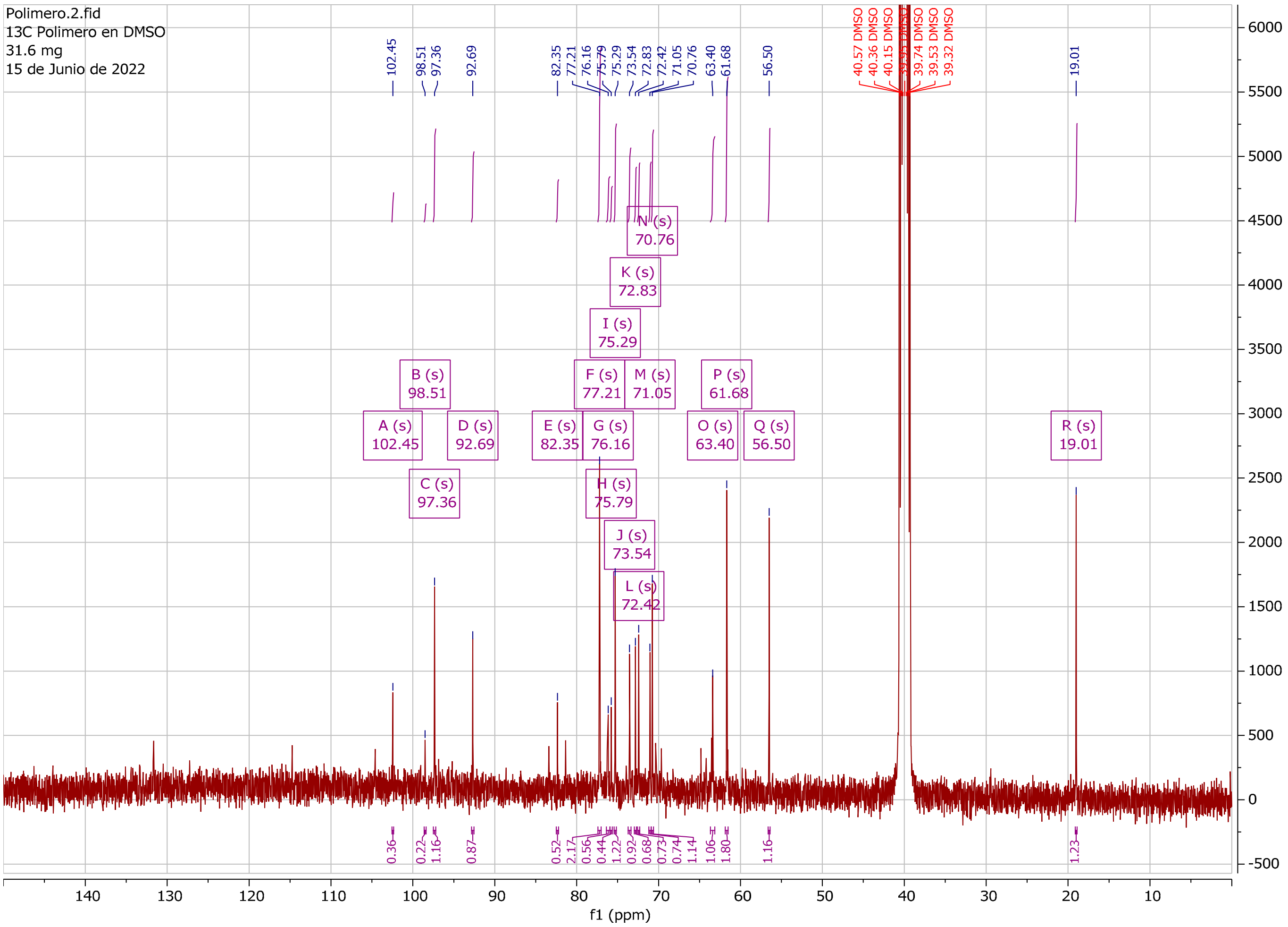

Figure S2. ^{13}C NMR spectrum (in DMSO-*d6*) of a mucilage sample extracted from OFI fruit peels.

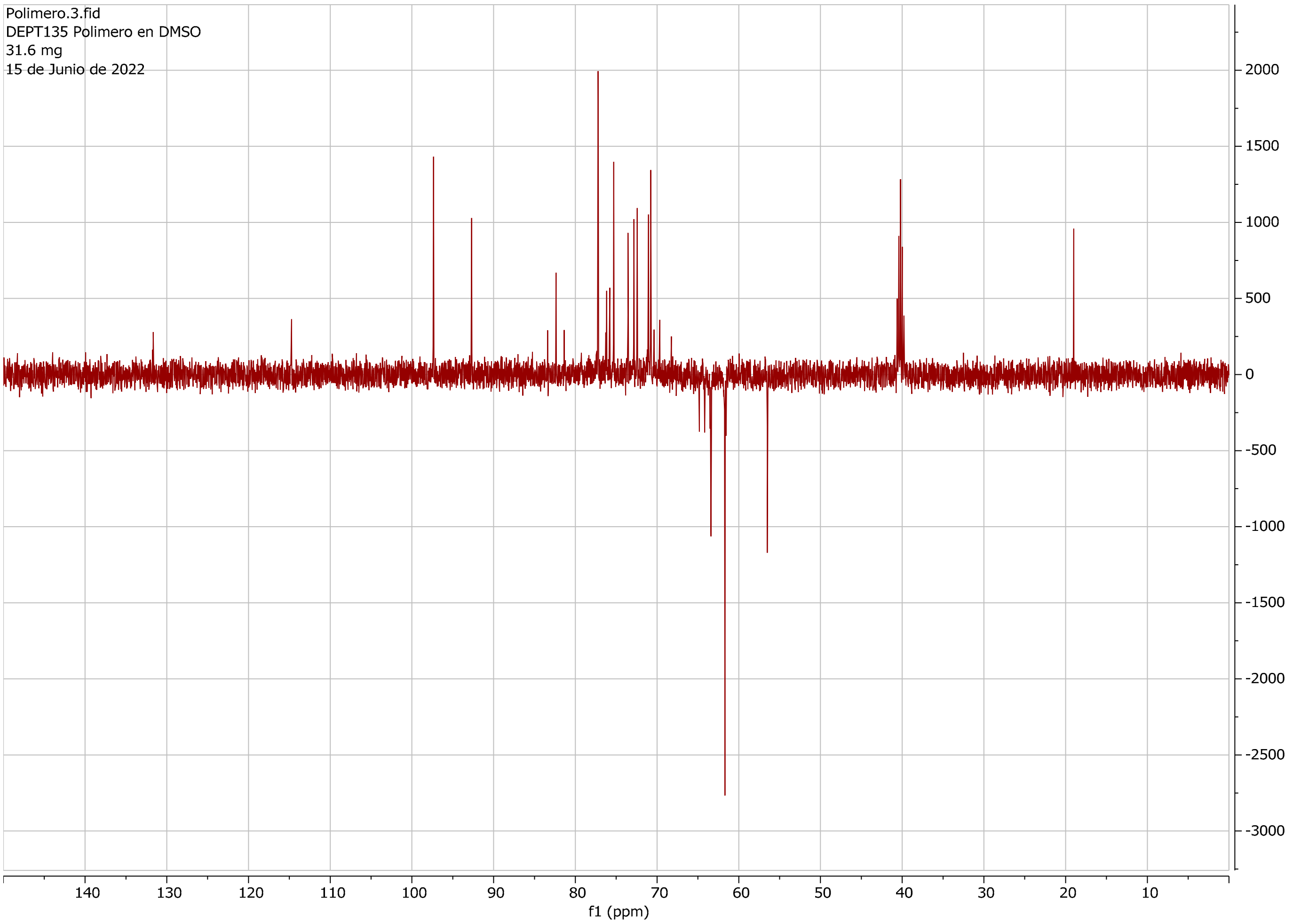

Figure S3. DEPT/^{13}C NMR spectrum (in DMSO-*d6*) of a mucilage sample extracted from OFI fruit peels.

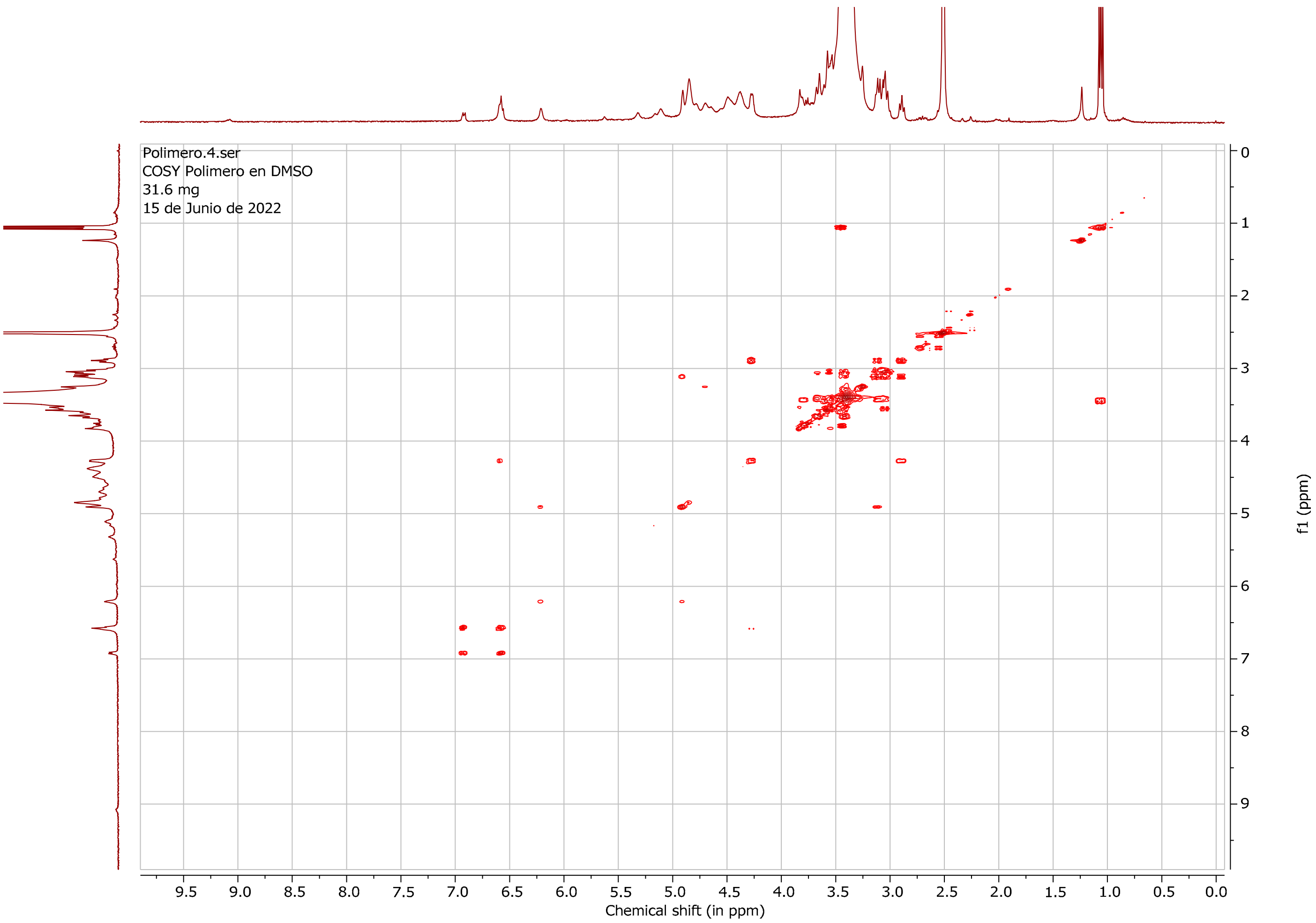

Figure S4. ^{1}H-^{1}H COSY NMR spectrum (in DMSO-*d6*) of a mucilage sample extracted from OFI fruit peels.

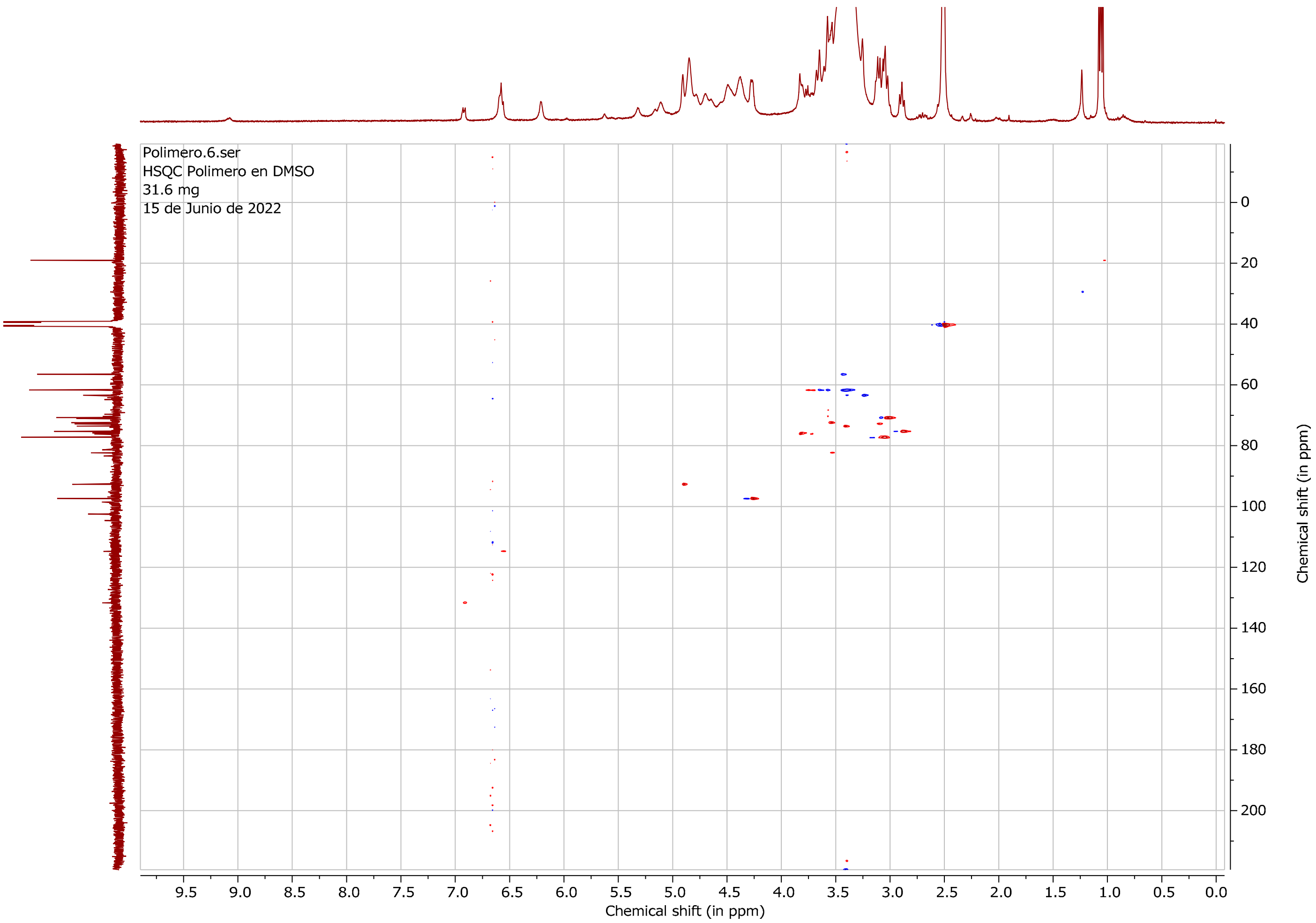

Figure S5. HSQC 2D-NMR spectrum (in DMSO-*d6*) of a mucilage sample extracted from OFI fruit peels.

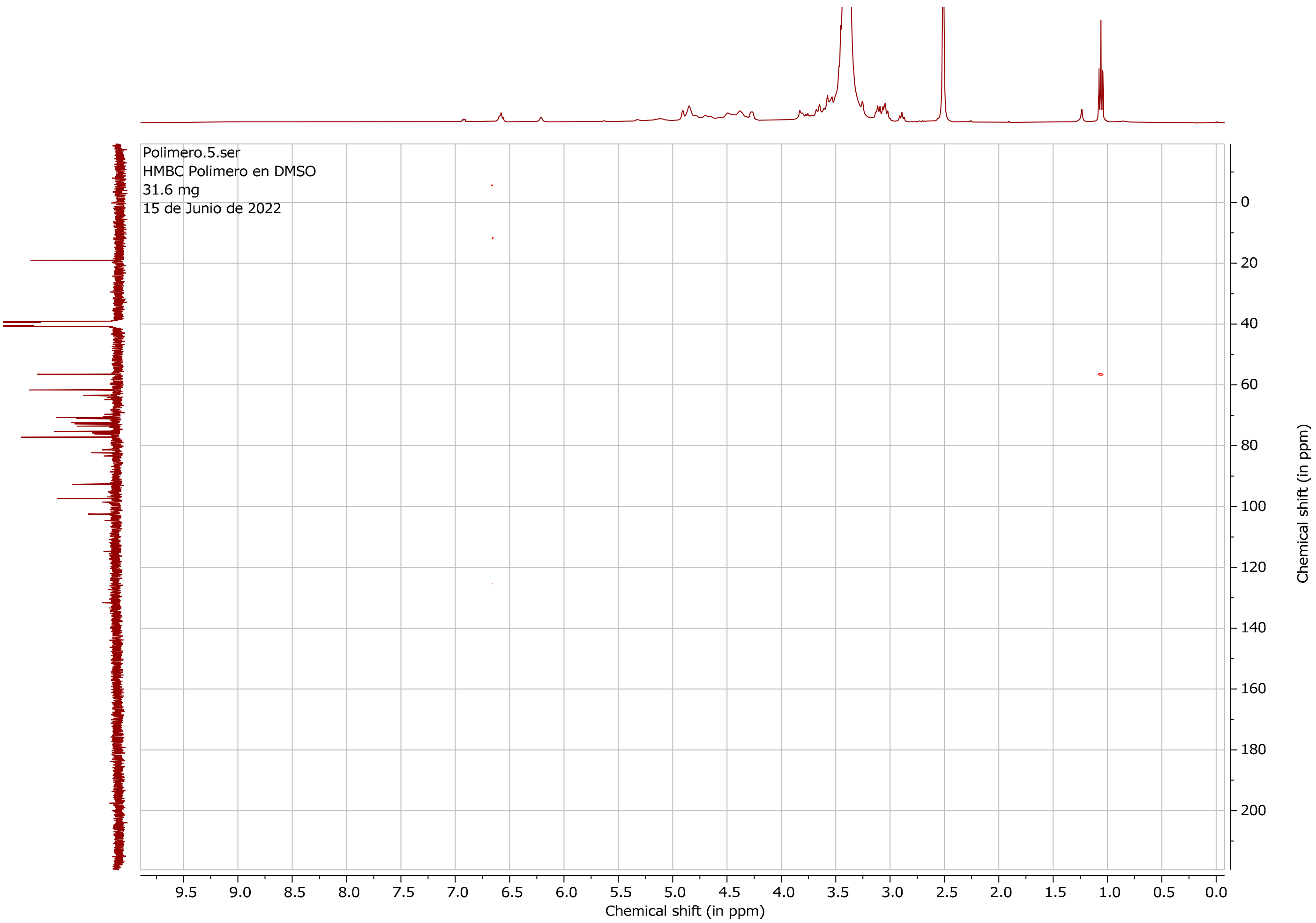

Figure S6. HMBC 2D-NMR spectrum (in DMSO-*d6*) of a mucilage sample extracted from OFI fruit peels.
